# Supplementary material for: Long-Term Mastication Changed Salivary Metabolomic Profiles
Source: Metabolites. 2022 Jul 18;12(7):660. doi: 10.3390/metabo12070660 (PMC9322701; doi:10.3390/metabo12070660)
Supplement: Supplementary file 1 [file metabolites-12-00660-s001.zip › metabolites-1772307-supplementary.pdf]

Supplementary Table S1. Salivary metabolites showing significant difference between PRE and POST samples

Metabolites	PRE		POST		F.C. (POST/PRE)	P-value	FDR	VIP score
	Mean	S.D.	Mean	S.D.				
o-Acetylcarnitine	0.187	0.110	0.0488	0.0455	0.26	$1.53×10^{-5}$	$1.55×10^{-4}$	1.7
Citrulline	7.99	9.11	2.49	2.63	0.31	$1.53×10^{-5}$	$1.55×10^{-4}$	1.2
Glutamine	15.5	8.93	5.31	3.87	0.34	$1.53×10^{-5}$	$1.55×10^{-4}$	1.5
Trimethylamine *N*-oxide	0.0684	0.0363	0.0234	0.00962	0.34	$1.53×10^{-5}$	$1.55×10^{-4}$	1.1
N-Acetylglucosamine	1.65	1.82	0.601	0.555	0.36	$1.53×10^{-5}$	$1.55×10^{-4}$	1.2
N^6,N^6,N^6-Trimethyllysine	0.0182	0.00913	0.00748	0.00386	0.41	$1.53×10^{-5}$	$1.55×10^{-4}$	1.9
Creatine	5.16	2.02	2.15	0.95	0.42	$1.53×10^{-5}$	$1.55×10^{-4}$	1.8
GABA	0.594	0.328	0.257	0.128	0.43	$3.05×10^{-5}$	$2.71×10^{-4}$	1.8
Guanosine	0.337	0.364	0.146	0.139	0.43	$7.62×10^{-5}$	$6.02×10^{-4}$	0.77
2-Oxoglutarate	0.894	0.406	0.396	0.232	0.44	$1.07×10^{-4}$	$6.32×10^{-4}$	1.8
Arginine	9.80	5.56	4.64	2.54	0.47	$1.07×10^{-4}$	$6.32×10^{-4}$	1.5
Glutamate	13.4	4.13	6.47	4.05	0.48	$1.07×10^{-4}$	$6.32×10^{-4}$	1.5
Creatinine	4.89	1.55	2.37	0.750	0.48	$1.52×10^{-4}$	$8.33×10^{-4}$	1.2
Indoleacetate	0.348	0.414	0.172	0.177	0.50	$2.14×10^{-4}$	0.0010	0.70
Alanine	12.5	10.1	6.35	3.56	0.51	$2.14×10^{-4}$	0.0010	0.56
Gluconate	0.639	0.359	0.327	0.191	0.51	$2.90×10^{-4}$	0.0012	1.1
Asparagine	1.18	1.07	0.604	0.282	0.51	$2.90×10^{-4}$	0.0012	0.63
Nicotinamide	0.181	0.116	0.0982	0.0558	0.54	$3.81×10^{-4}$	0.0015	0.67
Guanine	0.497	0.344	0.273	0.248	0.55	$5.48×10^{-4}$	0.0020	1.2
N-Acetylputrescine	0.431	0.475	0.240	0.162	0.56	$6.56×10^{-4}$	0.0022	0.59
Serine	5.49	2.99	3.09	1.18	0.56	$6.56×10^{-4}$	0.0022	0.44
b-Alanine	2.40	1.03	1.39	0.597	0.58	$8.39×10^{-4}$	0.0026	1.42
7-Methylguanine	0.0466	0.0189	0.0277	0.00893	0.59	$8.39 × 10^{-4}$	0.0026	0.62
Adenine	0.402	0.313	0.243	0.216	0.60	0.0011	0.0030	0.75

5-Aminopentanoate	82.0	62.4	49.6	40.8	0.61	0.0011	0.0030	0.15
Histidine	7.53	4.80	4.58	3.03	0.61	0.0013	0.0037	0.22
Hydroxyproline	0.859	0.468	0.523	0.455	0.61	0.0026	0.0068	0.95
Proline	15.8	17.7	9.66	13.9	0.61	0.0032	0.0080	0.59
Adenosine	0.236	0.100	0.145	0.0627	0.61	0.0045	0.011	0.67
Choline	1.70	0.779	1.10	0.527	0.65	0.0046	0.011	1.2
Aspartate	7.03	3.16	4.58	2.19	0.65	0.0046	0.011	0.02
Tryptophan	0.223	0.138	0.146	0.0896	0.65	0.0056	0.012	0.34
Tyrosine	10.3	5.58	6.76	4.68	0.66	0.0079	0.017	0.22
Taurine	15.6	7.93	10.4	4.48	0.67	0.0093	0.020	0.40
3-Methylguanine	0.0335	0.0143	0.0255	0.0117	0.76	0.011	0.022	0.13
Carnitine	0.753	0.356	0.579	0.193	0.77	0.011	0.022	0.37
Phenylalanine	4.87	3.08	3.79	3.22	0.78	0.015	0.029	0.30
Glycolate	3.11	1.76	4.51	2.20	1.5	0.017	0.031	1.5
N^1-Acetylspermine	0.0169	0.00507	0.0272	0.00560	1.6	0.023	0.040	1.6
Succinate	2.85	1.86	4.69	3.13	1.6	0.023	0.040	1.6
2-Hydroxyglutarate	0.512	0.305	1.42	2.03	2.8	0.023	0.040	1.3
Citrate	1.58	1.13	5.78	4.03	3.7	0.031	0.0504	1.8
Hexylamine	0.139	0.0184	0.913	0.228	6.6	0.031	0.0504	1.9

S.D. and F.C. indicate standard deviation and fold change, respectively.

FDR indicate false dscovery rate-corrected P-value.

VIP score indicates variable importance in projection that was obtained from PLS-DA model.

Supplementary Table S2. The linearity range of standard mixture samples

Metabolites	Regression equation	slope	intercept	Liniarity(R^2)	Linear range (μmol/L)
**Positive**					
GABA	Y = 0.4151x +0.0103	0.415	0.01026	0.9971	0.01-1
Adenine	Y = 1.6055x +0.1929	1.605	0.19293	0.9967	0.01-10
Adenosine	Y = 2.4149x +0.3217	2.415	0.32173	0.9960	0.001-10
Anthranilate	Y = 0.7222x -0.0187	0.722	-0.01866	0.9978	0.01-10
Arginine	Y = 1.0084x +0.1042	1.008	0.10418	0.9970	0.003-10
N-Acetylputrescine	Y = 1.7444x +0.1922	1.744	0.19223	0.9985	0.001-30
6-Aminohexanoate	Y = 1.1233x +0.0166	1.123	0.01658	0.9983	0.01-30
N-Acetylhistidine	Y = 1.0135x +0.0803	1.013	0.08032	0.9977	0.003-10
N-Acetylhistamine	Y = 3.6034x +0.3633	3.603	0.36329	0.9958	0.001-10
Agmatine	Y = 4.3211x +0.0151	4.321	0.01509	0.9994	0.001-1
2-Aminoadipate	Y = 0.6272x +0.0248	0.627	0.02476	0.9981	0.001-1
N1-Acetylspermine	Y = 0.6755x +0.0109	0.676	0.01089	0.9987	0.001-10
N8-Acetylspermidine	Y = 0.535x +0.1189	0.535	0.11891	0.9965	0.001-30
N-Acetylglucosamine	Y = 0.7161x +0.0207	0.716	0.02068	0.9991	0.003-3
5-Aminopentanoate	Y = 0.5277x +0.2157	0.528	0.21570	0.9983	0.1-30
O-Acetylcarnitine	Y = 6.0668x +0.1962	6.067	0.19620	0.9995	0.001-10
N-Epsilon-Acetyllysine	Y = 0.9048x -0.2267	0.905	-0.22668	0.9964	0.003-30
ADMA	Y = 1.0699x +0.0826	1.070	0.08261	0.9958	0.003-30
3-Aminoisobutanoate	Y = 0.4525x +0.0179	0.453	0.01795	0.9980	0.01-3
N1-Acetylspermidine	Y = 0.8946x -0.0188	0.895	-0.01884	0.9969	0.001-10
Choline	Y = 5.7929x +0.0395	5.793	0.03950	0.9995	0.003-3
Creatine	Y = 1.3479x +0.0351	1.348	0.03508	0.9951	0.003-1
Cytidine	Y = 0.2799x -0.0123	0.280	-0.01229	0.9984	0.03-3
Cadaverine	Y = 0.768x +0.0066	0.768	0.00665	0.9986	0.01-0.3

Cytosine	Y = 0.6343x +0.0242	0.634	0.02424	0.9977	0.01-3
Creatinine	Y = 1.0966x +0.0327	1.097	0.03272	0.9978	0.003-3
Carnitine	Y = 4.8275x +0.0449	4.828	0.04493	0.9967	0.003-1
gamma-Butyrobetaine	Y = 4.5884x +0.064	4.588	0.06400	0.9981	0.001-3
Diethanolamine	Y = 2.4829x +0.0247	2.483	0.02474	0.9989	0.01-0.3
Deoxycytidine	Y = 0.0148x -0.0021	0.015	-0.00213	0.9965	0.1-10
5'-Methylthioadenosine	Y = 3.1492x +0.2799	3.149	0.27989	0.9962	0.001-10
Deoxyinosine	Y = 1.2253x +0.0217	1.225	0.02175	0.9968	0.003-1
N1,N8-Diacetylspermidine	Y = 1.1824x -0.0328	1.182	-0.03278	0.9978	0.001-3
N1,N12-Diacetylspermine	Y = 0.9334x -0.0343	0.933	-0.03432	0.9964	0.001-3
Oxiglutatione	Y = 0.0999x -0.0008	0.100	-0.00083	0.9983	0.03-1
Guanine	Y = 0.7003x +0.1135	0.700	0.11349	0.9975	0.01-30
Guanosine	Y = 0.3866x -0.0112	0.387	-0.01120	0.9978	0.01-10
4-Guanidinobutanoate	Y = 1.7643x +0.2271	1.764	0.22711	0.9970	0.001-10
Glucosamine	Y = 0.2479x +0.0019	0.248	0.00189	0.9969	0.01-0.3
Histidine	Y = 0.3459x +0.0204	0.346	0.02036	0.9968	0.01-3
Hypoxanthine	Y = 3.8119x -0.0501	3.812	-0.05006	0.9995	0.003-10
Hexylamine	Y = 2.2422x +0.2284	2.242	0.22843	0.9958	0.003-10
Histamine	Y = 1.9954x +0.0051	1.995	0.00510	0.9995	0.003-0.3
Hydroxylysine	Y = 0.4358x +0.0246	0.436	0.02457	0.9960	0.01-3
Inosine	Y = 0.1477x -0.056	0.148	-0.05598	0.9953	0.03-30
Indoleacetate	Y = 0.5212x +0.0271	0.521	0.02713	0.9963	0.01-3
Imidazoleacetate	Y = 0.8581x +0.0394	0.858	0.03936	0.9978	0.01-3
Leucine	Y = 1.2707x -0.0711	1.271	-0.07105	0.9996	0.01-10
Lysine	Y = 0.3681x +0.02	0.368	0.02004	0.9953	0.003-3
Methionine	Y = 0.6653x -0.019	0.665	-0.01901	0.9977	0.01-30
1-Methylhistamine	Y = 1.6756x +0.4213	1.676	0.42135	0.9979	0.003-30
Metformin	Y = 4.6789x +0.0979	4.679	0.09791	0.9974	0.003-3
3-Methylguanine	Y = 0.8233x +0.0992	0.823	0.09917	0.9985	0.01-30

7-Methylguanine	Y = 1.2501x +0.0318	1.250	0.03183	0.9986	0.001-3
1-Methyladenosine	Y = 3.2986x +0.0935	3.299	0.09355	0.9962	0.001-3
1-Methylnicotinamide	Y = 5.187x +0.0597	5.187	0.05967	0.9962	0.001-1
N-Methylaniline	Y = 4.2528x -0.0517	4.253	-0.05167	0.9999	0.003-3
3-Methylhistidine	Y = 0.7127x +0.0214	0.713	0.02144	0.9975	0.003-3
Nicotinamide	Y = 0.6158x +0.029	0.616	0.02898	0.9968	0.003-3
Nicotine	Y = 3.1544x +0.1142	3.154	0.11416	0.9968	0.003-3
N6,N6,N6-Trimethyllysine	Y = 1.4937x +0.0893	1.494	0.08929	0.9983	0.001-10
Ornithine	Y = 0.2275x +0.0072	0.228	0.00723	0.9968	0.01-1
Ophthalmate	Y = 0.8365x -0.3259	0.836	-0.32594	0.9955	0.01-30
Phenylalanine	Y = 1.6136x -0.068	1.614	-0.06798	0.9996	0.003-10
Proline	Y = 1.2221x +0.0273	1.222	0.02727	0.9996	0.01-10
Pipecolate	Y = 1.7392x -0.0637	1.739	-0.06373	0.9998	0.003-10
Pyridoxamine	Y = 2.3173x +0.0035	2.317	0.00348	0.9985	0.003-0.1
Spermidine	Y = 1.064x +0.2046	1.064	0.20465	0.9964	0.003-30
Spermine	Y = 0.8628x +0.0088	0.863	0.00877	0.9959	0.01-10
SDMA	Y = 1.1824x -0.0328	1.182	-0.03278	0.9978	0.001-3
Tyrosine	Y = 0.5199x -0.0775	0.520	-0.07752	0.9977	0.01-30
Tryptophan	Y = 1.089x -0.2607	1.089	-0.26070	0.9976	0.01-30
Tyramine	Y = 0.6267x -0.0278	0.627	-0.02780	0.9975	0.003-30
Thymine	Y = 0.8581x +0.0394	0.858	0.03936	0.9978	0.01-3
Thiamine	Y = 5.215x +0.0719	5.215	0.07189	0.9970	0.003-1
Uracil	Y = 0.038x +0.0023	0.038	0.00231	0.9979	0.1-3
Urocanate	Y = 0.5332x +0.1991	0.533	0.19909	0.9950	0.01-30
Valine	Y = 0.9887x -0.1574	0.989	-0.15742	0.9980	0.3-30
Xanthine	Y = 0.1307x +0.0042	0.131	0.00421	0.9961	0.1-1
Alanine	Y = 0.2367x +0.0062	0.237	0.00620	0.9989	0.01-3
Asparagine	Y = 0.2605x +0.0099	0.260	0.00994	0.9994	0.01-10
Aspartate	Y = 0.2064x +0.0294	0.206	0.02936	0.9965	0.01-10

S-Adenosylmethionine	Y = 0.4795x +0.0279	0.479	0.02788	0.9995	0.003-10
beta-Alanine	Y = 0.249x +0.0164	0.249	0.01643	0.9960	0.01-3
S-Adenosylhomocysteine	Y = 0.2561x -0.0124	0.256	-0.01238	0.9966	0.01-3
alpha-Aminobutyrate	Y = 0.2552x +0.0096	0.255	0.00959	0.9961	0.03-3
Putrescine	Y = 0.4779x +0.0113	0.478	0.01134	0.9973	0.01-1
Carnosine	Y = 0.6388x -0.1042	0.639	-0.10420	0.9951	0.01-10
Citrulline	Y = 0.3771x -0.0192	0.377	-0.01921	0.9987	0.01-30
Cysteine	Y = 0.2783x +0.0018	0.278	0.00175	0.9996	0.01-3
Cystathionine	Y = 0.2281x +0.0688	0.228	0.06879	0.9955	0.01-30
Levodopa	Y = 0.5322x +0.0146	0.532	0.01457	0.9970	0.003-3
Glutathione	Y = 0.4707x +0.0073	0.471	0.00727	0.9984	0.01-30
Glycine	Y = 0.1275x +0.0083	0.127	0.00834	0.9966	0.03-3
Glutamine	Y = 0.5233x +0.0107	0.523	0.01072	0.9984	0.003-30
Glutamate	Y = 0.4496x +0.0062	0.450	0.00624	0.9997	0.003-3
Homoserine	Y = 1.0702x -0.0051	1.070	-0.00512	0.9999	0.003-3
Hydroxyproline	Y = 0.765x +0.161	0.765	0.16101	0.9960	0.01-30
Homocysteine	Y = 0.4931x +0.0281	0.493	0.02806	0.9954	0.01-3
Hypotaurine	Y = 0.3471x -0.0253	0.347	-0.02530	0.9984	0.1-30
Isoleucine	Y = 1.2159x -0.0448	1.216	-0.04483	0.9999	0.03-10
Serine	Y = 0.1994x +0.0086	0.199	0.00860	0.9958	0.01-3
Taurine	Y = 0.0739x +0.0347	0.074	0.03473	0.9956	0.1-30
Threonine	Y = 1.0702x -0.0051	1.070	-0.00512	0.9999	0.003-3
Uridine	Y = 0.0195x +0.0309	0.019	0.03092	0.9809	0.03-30
Trimethylamine N-oxide	Y = 2.505x +0.0126	2.505	0.01255	0.9978	0.003-3
**Negative**					
AMP	Y = 0.0848x +0.0027	0.085	0.00266	0.9971	0.003-3
ADP	Y = 0.0894x -0.007	0.089	-0.00698	0.9917	1-10
cis-Aconitate	Y = 0.103x -0.0168	0.103	-0.01683	0.9852	0.1-30

cAMP	Y = 0.1279x +0.0071	0.128	0.00708	0.9985	0.003-10
Citrate	Y = 0.0619x +0.0026	0.062	0.00258	0.9963	0.01-3
CMP	Y = 0.0584x +0.0009	0.058	0.00090	0.9972	0.01-1
3-Sulfinoalanine	Y = 0.0059x +0.001	0.006	0.00098	0.9658	0.03-3
CDP	Y = 0.0745x +0.002	0.075	0.00202	0.9954	0.03-3
NADH	Y = 0.0133x -0.00003	0.013	-0.00003	0.9940	0.3-10
NAD+	Y = 0.0105x +0.0017	0.010	0.00171	0.9980	0.3-3
Dihydroxyacetone phosphate	Y = 0.0218x +0.0044	0.022	0.00442	0.9852	0.3-3
Fructose 6-phosphate	Y = 0.2452x +0.0038	0.245	0.00384	0.9958	0.01-1
FAD	Y = 0.0949x +0.0007	0.095	0.00071	0.9989	0.03-10
Fumarate	Y = 0.0092x +0.0001	0.009	0.00009	0.9937	0.1-1
Fructose 1,6-bisphosphate	Y = 0.0483x +0.0112	0.048	0.01125	0.9691	0.3-10
Glucose 6-phosphate	Y = 0.2452x +0.0038	0.245	0.00384	0.9958	0.01-1
Glycolate	Y = 0.0235x -0.0077	0.024	-0.00775	0.9953	0.3-10
Glyoxylate	Y = 0.0115x -0.0011	0.011	-0.00110	0.9954	0.1-10
GMP	Y = 0.0817x +0.0024	0.082	0.00240	0.9961	0.003-3
Glucose 1-phosphate	Y = 0.2452x +0.0038	0.245	0.00384	0.9958	0.01-1
GDP	Y = 0.0535x +0.1552	0.053	0.15524	0.9048	3-30
Glyceraldehyde 3-phosphate	Y = 0.0218x +0.0044	0.022	0.00442	0.9852	0.3-3
Glycerol 3-phosphate	Y = 0.0533x +0.002	0.053	0.00197	0.9982	0.01-1
Gluconate	Y = 0.0728x +0.0021	0.073	0.00214	0.9968	0.01-1
cGMP	Y = 0.1045x +0.0148	0.105	0.01482	0.9955	0.01-10
2-Hydroxyglutarate	Y = 0.0507x +0.004	0.051	0.00403	0.9952	0.01-10
Isocitrate	Y = 0.0479x +0.0039	0.048	0.00389	0.9950	0.03-1
IMP	Y = 0.1x +0.0033	0.100	0.00335	0.9967	0.01-3
Lactate	Y = 0.0214x +0.0031	0.021	0.00313	0.9959	0.1-3
Malate	Y = 0.0372x +0.0004	0.037	0.00038	0.9955	0.03-1
3-Methyl-2-oxobutanoate	Y = 0.0323x +0.0054	0.032	0.00540	0.9966	0.1-10
NADP+	Y = 0.0453x -0.0014	0.045	-0.00140	0.9983	0.1-3

2-Oxoglutarate	Y = 0.0239x -0.0009	0.024	-0.00093	0.9994	0.1-10
Phosphoenolpyruvate	Y = 0.0283x -0.0033	0.028	-0.00325	0.9947	0.3-30
Pyruvate	Y = 0.0096x +0.0014	0.010	0.00143	0.9618	1-3
3-Phosphoglycerate	Y = 0.0671x +0.0007	0.067	0.00066	0.9951	0.1-3
2-Phosphoglycerate	Y = 0.0671x +0.0007	0.067	0.00066	0.9951	0.1-3
6-Phosphogluconate	Y = 0.0721x +0.0001	0.072	0.00013	0.9960	0.01-1
Ribose 5-phosphate	Y = 0.1391x -0.0011	0.139	-0.00114	0.9965	0.03-0.3
Ribulose 5-phosphate	Y = 0.1391x -0.0011	0.139	-0.00114	0.9965	0.03-0.3
Succinate	Y = 0.0394x +0.0017	0.039	0.00167	0.9964	0.03-1
Sedoheptulose 7-phosphate	Y = 0.0932x -0.00001	0.093	-0.00001	0.9975	0.01-0.3
dTDP	Y = 0.0407x +0.4928	0.041	0.49285	0.9613	10-100
dTMP	Y = 0.1522x +0.003	0.152	0.00302	0.9989	0.01-3
Erythrose 4-phosphate	Y = 0.0214x +0.0007	0.021	0.00070	0.9910	0.01-1

Substances that could not be separated are shown with the same color.

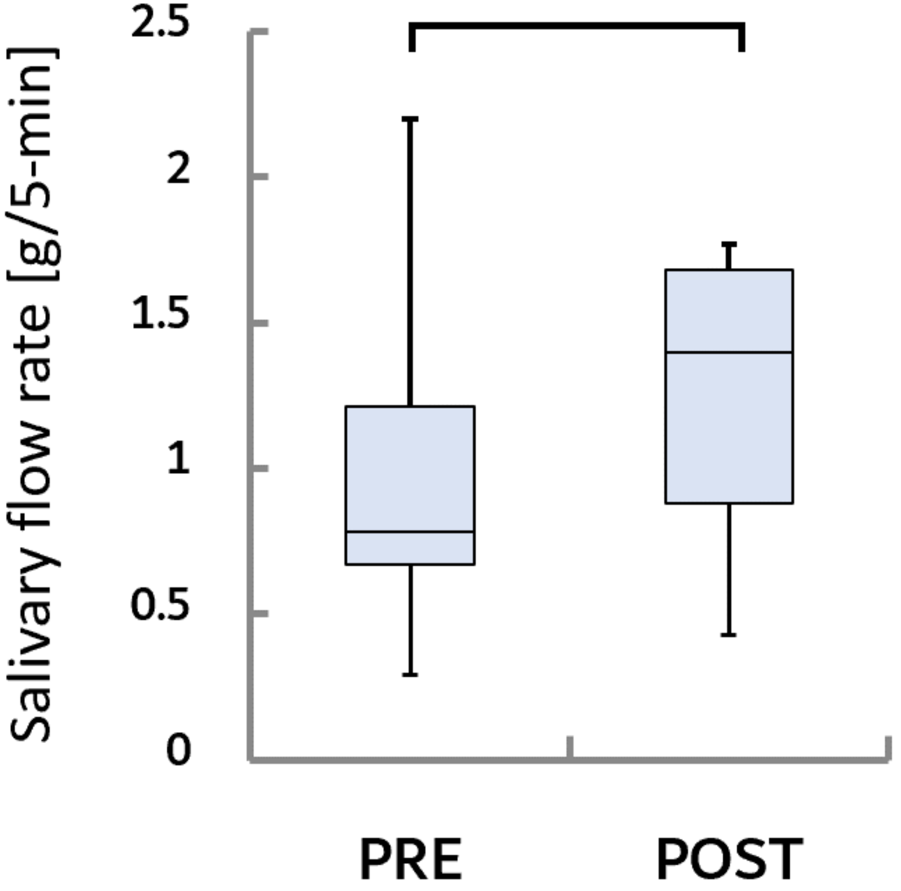

Supplementary Figure S1. Change in unstimulated salivary flow rate
The unstimulated salivary flow rate was evaluated using a 5-min spitting procedure. $P<0.05$, Wilcoxon signed-rank test.

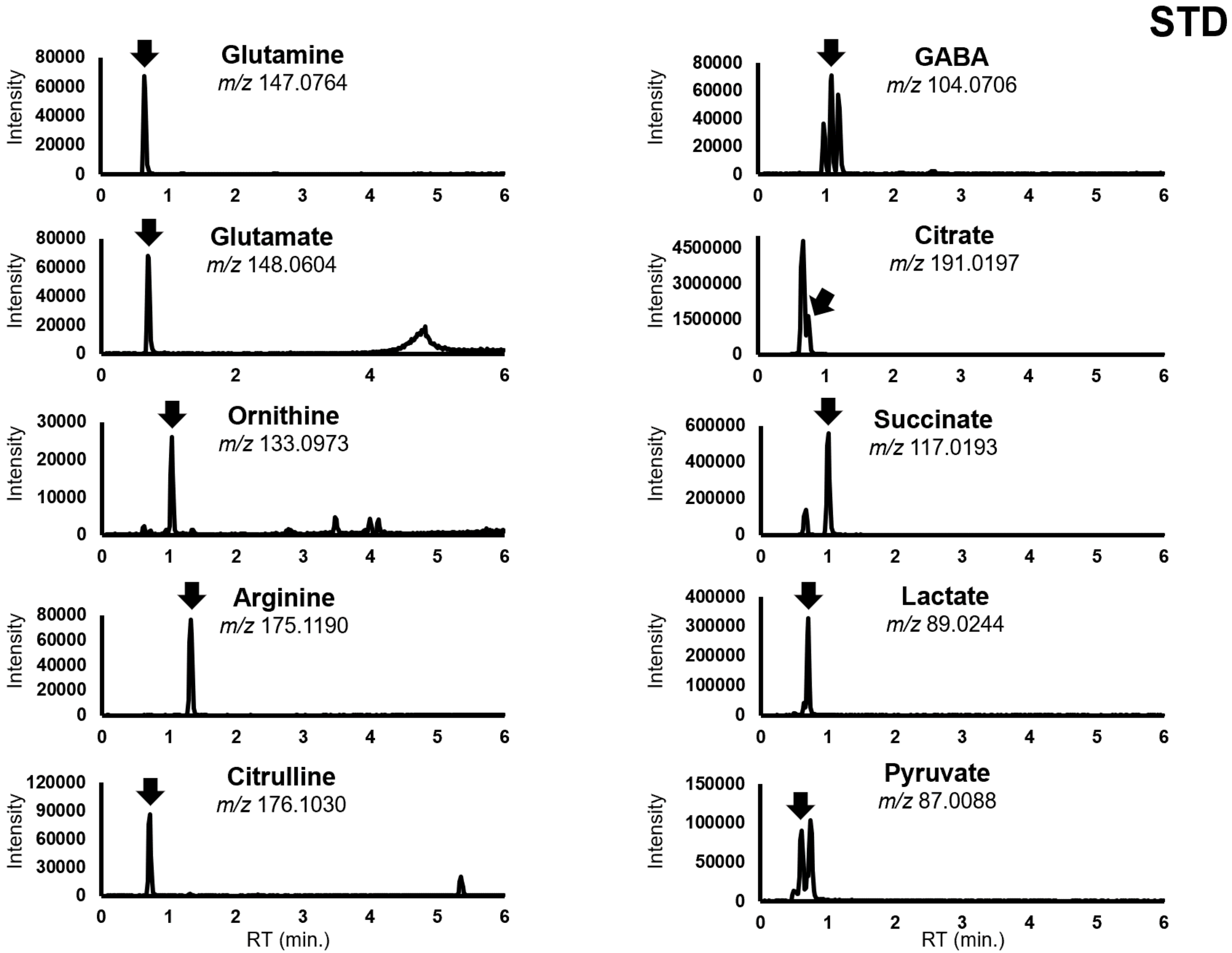

Supplementary Figures S2-1. Representative LCMS chromatogram of 10 metabolites (in standard mixture samples)

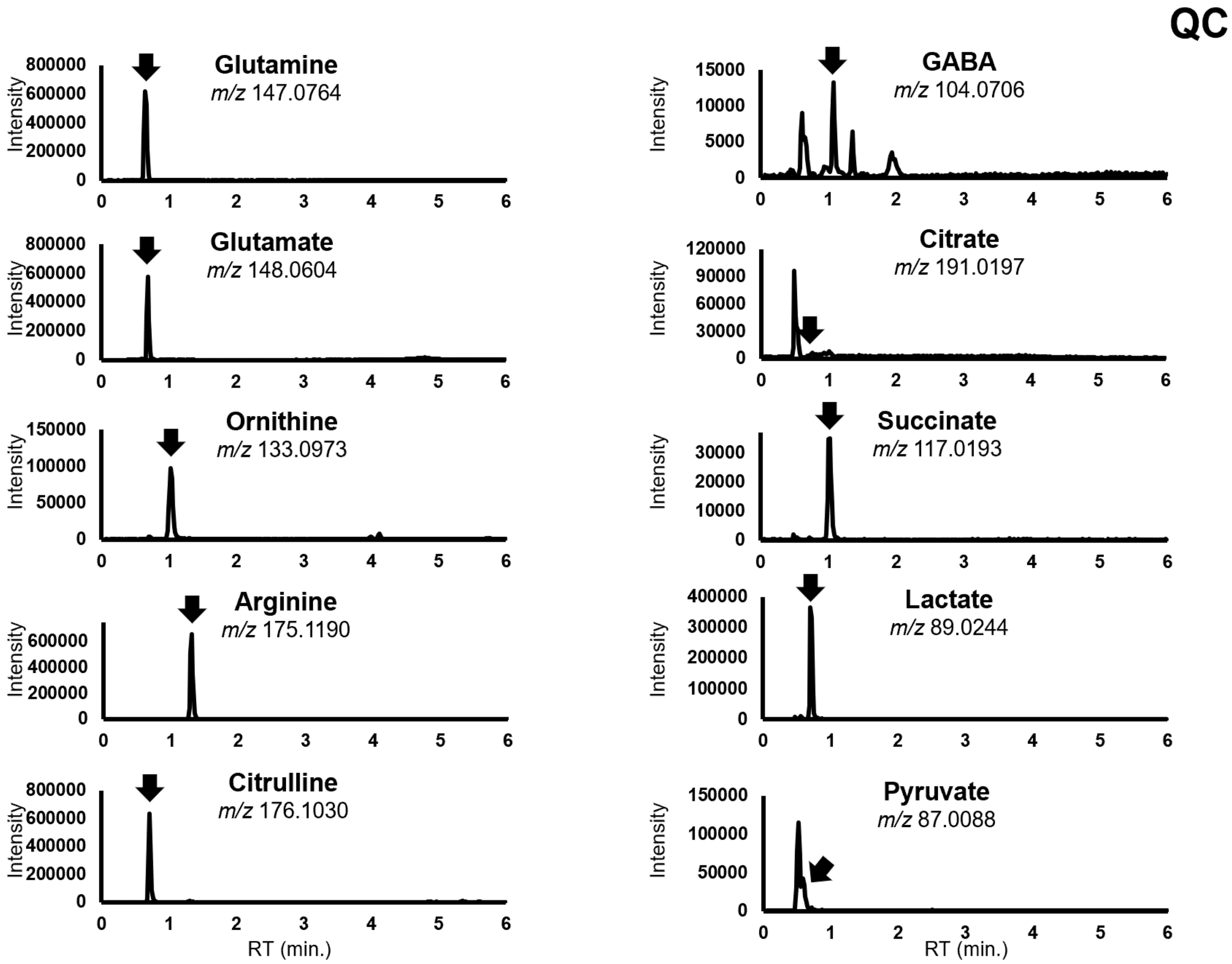

Supplementary Figures S2-2. Representative LCMS chromatogram of 10 metabolites (in quality control samples)
